# Underrepresentation of women in randomized controlled trials: a systematic review and meta-analysis

**DOI:** 10.1186/s13063-022-07004-2

**Published:** 2022-12-21

**Authors:** Vered Daitch, Adi Turjeman, Itamar Poran, Noam Tau, Irit Ayalon-Dangur, Jeries Nashashibi, Dafna Yahav, Mical Paul, Leonard Leibovici

**Affiliations:** 1 Department of Medicine E, Rabin Medical Center, Beilinson Hospital, 39 Jabotinski Road, 49100 Petah Tikva, Israel; 2 Sackler Faculty of Medicine, Tel Aviv University, Tel Aviv, Israel; 3 Department of Diagnostic Imaging, Sheba Medical Center, Ramat Gan, Israel; 4 Department of Internal Medicine D, Rambam Health Care Campus, Haifa, Israel; 5 Infectious Diseases Unit, Rabin Medical Center, Beilinson Hospital, Petah Tikva, Israel; 6 Infectious Diseases Institute, Rambam Health Care Campus, Haifa, Israel; 7 The Ruth and Bruce Rappaport Faculty of Medicine, Technion-Israel Institute of Technology, Haifa, Israel

**Keywords:** Population external validity, Underrepresentation of women, Randomized controlled trials

## Abstract

**Background:**

Although regulatory changes towards correcting the underrepresentation of women in randomized controlled trials (RCTs) occurred (National Institutes of Health 1994), concerns exist about whether an improvement is taking place. In this systematic review and meta-analysis, we aimed to assess the inclusion rates of women in recent RCTs and to explore the potential barriers for the enrollment of women.

**Methods:**

RCTs published in 2017 examining any type of intervention in adults were searched in PubMed and Cochrane Library. The following predefined medical fields were included: cardiovascular diseases, neoplasms, endocrine system diseases, respiratory tract diseases, bacterial and fungal infections, viral diseases, digestive system diseases, and immune system diseases. Studies were screened independently by two reviewers, and an equal number of studies was randomly selected per calendric month. The primary outcome was the enrollment rate of women, calculated as the number of randomized women patients divided by the total number of randomized patients. Rates were weighted by their inverse variance; statistical significance was tested using general linear models (GLM).

**Results:**

Out of 398 RCTs assessed for eligibility, 300 RCTs were included. The enrollment rate of women in all the examined fields was lower than 50%, except for immune system diseases [median enrollment rate of 68% (IQR 46 to 81)]. The overall median enrollment rate of women was 41% (IQR 27 to 54). The median enrollment rate of women decreased with older age of the trials’ participants [mean age of trials’ participants ≤ 45 years: 47% (IQR 30–64), 46–55 years: 46% (IQR 33–58), 56–62 years: 38% (IQR 27–50), ≥ 63 years: 33% (IQR 20–46), *p* < 0.001]. Methodological quality characteristics showed no significant association with the enrollment rates of women. Out of the 300 included RCTs, eleven did not report on the number of included women. There was no significant difference between these studies and the studies included in the analysis.

**Conclusions:**

Women are being inadequately represented, in the selected medical fields analyzed in our study, in recent RCTs. Older age is a potential barrier for the enrollment of women in clinical trials. Low inclusion rates of elderly women might create a lack of crucial knowledge in the adverse effects and the benefit/risk profile of any given treatment. Factors that might hinder the participation of women should be sought and addressed in the design of the study.

**Supplementary Information:**

The online version contains supplementary material available at 10.1186/s13063-022-07004-2.

## Introduction

Population external validity of a randomized controlled trial (RCT) is defined as the extent to which the results of a trial can be generalized from a specific sample to a target population [1]. For many years, the population external validity of RCTs was compromised due to the inclusion of mainly male participants, while women were underrepresented [2, 3]. When applying gender-unbalanced RCTs to real-life clinical settings, concerns arise as treatment dosing and effects may not be similar between the predominantly male RCT population and women patients [2, 3]. Drug effects may vary between the sexes according to body composition and size and pharmacokinetic or pharmacodynamic parameters [3, 4]. This may lead to inappropriate dosing and inaccurate estimation of side effects in women and, ultimately, to overall less qualitative patient care and suboptimal clinical treatment outcomes for women patients.

The most significant change in the US Food and Drug Administration’s (FDA) regulations towards correcting the underrepresentation of women occurred in 2000. This particular regulation permits the FDA to place a clinical hold on investigational new drug studies for the treatment of a serious or life-threatening disease if women or men are excluded from a clinical trial due to reproductive potential [5]. This change was reinforced by an audit performed in 2001 by the US Government Accountability Office, which found that eight out of ten drugs withdrawn from the US market between 1997 and 2001 had more severe adverse events in women than in men, largely because these drugs were not sufficiently tested on women [6]. Another significant milestone is the EU Clinical Trials Regulation No 536/2014. This regulation lists specific population groups that are likely to use the investigated medicinal product, to be included in the clinical trial. This new legal addition contains provisions for including pregnant and breastfeeding women in clinical trials [7].

Pivotal trials and studies from several medical fields (cardiovascular diseases, HIV, stroke, and cancer) show that the change put forward by these regulations has yet to come [8–16]. There is a gap in information for many major health burden medical conditions. This has led us to perform a systematic review and meta-analysis of the literature to assess the inclusion rates of women in recently published randomized controlled trials and to further explore the potential barriers to enrollment of women.

## Methods

We included randomized controlled trials in the following Medical Subject Headings (MESH) categories: cardiovascular diseases, neoplasms, endocrine system diseases, respiratory tract diseases, bacterial and fungal infections, viral diseases, digestive system diseases, and immune system diseases. These areas were chosen due to their major health burden in terms of disability and death [17]. We included any type of intervention in adults (age ≥ 18 years).

We conducted a comprehensive search to identify all RCTs published during 2017 in PubMed and Cochrane Library. Our full search phrase is presented in Additional file 1: Appendix 1. Out of 26,994 identified records, we have randomly selected and screened 1098 records. The function “RAND” in Excel was used, a unique random number was assigned to each trial, numbers were sorted from smallest to largest. An equal number of studies per calendric month was reviewed by two investigators (AT, IP). Records were excluded if they were duplicates, not RCTs, not written in English, included patients under the age of 18, and if they examined a sex-specific condition. Sex-specific condition was defined as a condition that occurs only in people of one sex, such as prostate cancer, ovarian cancer, pregnancy and delivery-related conditions, endometriosis, polycystic ovary syndrome, and bacterial vaginosis.

For each included study, we extracted data on the main disease or disorder, funding, patient characteristics (age, gender), hypothesis, intervention type, setting, countries (developed and developing economies), centers, study duration, follow-up duration, number of screened patients, number of randomized patients, methodological characteristics (allocation concealment, blinding), and study endpoints (soft, surrogate or hard outcome, outcome of primary hypothesis).

Country classifications to developed and developing economies were performed according to the United Nations’ “World Economic Situation and Prospects 2022” statistical annex [18].

Soft outcomes were defined as patient-reported outcomes and symptomatology. Surrogate outcomes were defined as a laboratory measure or physical sign that is intended to be used as a substitute for a clinical endpoint that matters to patients. Hard outcomes were defined as acute coronary syndrome and stroke, pathological diagnosis, overall survival, and mortality [19].

The risk of bias was assessed by both investigators according to the Cochrane Handbook for Systematic Reviews of Interventions [20].

Our primary outcome was the enrollment rate of women, calculated as the number of randomized women patients divided by the total number of randomized patients.

### Statistical analysis

Analysis was performed using the Statistical Package for the Social Sciences 27 (SPSS Inc.).

Data are presented as percentages for categorical variables, and as median and interquartile range (IQR, 25–75 percentiles) for non-normally distributed continuous variables. Associations between median enrollment rates and trial characteristics, and median enrollment rates and medical conditions were tested in a univariate analysis. Categorical data were compared using the chi-square test. For the meta-analysis, we weighted rates by the inverse variance. Statistical significance was tested using general linear models (GLM).

## Results

**Fig. 1:**
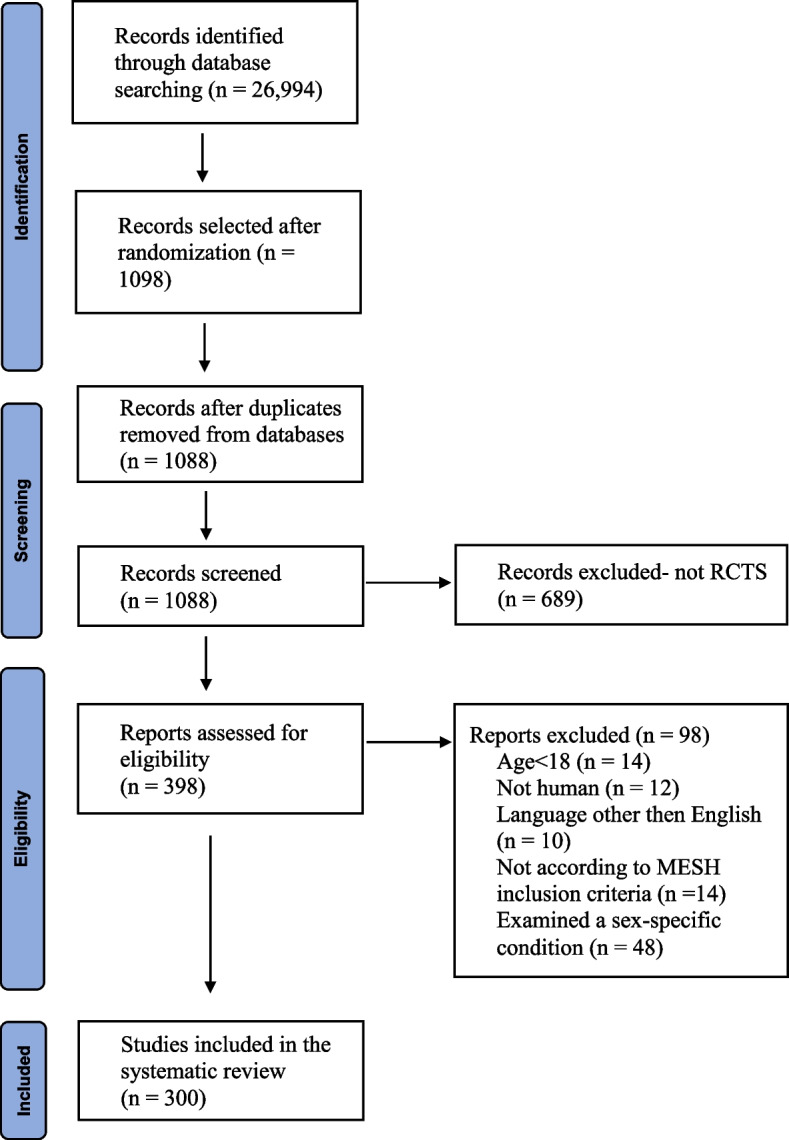
PRISMA flow diagram

Out of 398 RCTs (~33 each month) assessed for eligibility, 300 RCTs were included in this systematic review and meta-analysis (Fig. 1).

The enrollment rate of women in all the examined diseases was lower than 50%, except for immune system diseases. The overall median enrollment rate of women was 41% (IQR 27–54; *n* = 289). In Table 1, we present the median enrollment rates of women in trials examining different medical conditions. The lowest median enrollment rate of women was in trials examining ischemic heart disease [22% (IQR 17–33); *n* = 17]. Studies examining immune system diseases had the highest proportion of women [68% (46–81); *n* = 12]. Many of the trials (by field) recruited less than 40% women: HIV/AIDS [33% (IQR 9–57); *n* = 13], bacterial and fungal infections [37% (IQR 25–49); *n* = 24], congestive heart failure [34% (IQR 25–53); *n* = 14], hypertension [38% (19–52); *n* = 11], liver disease [35% (IQR 26–46); *n* = 12], neoplasms [39% (IQR 28–47); *n* = 53], type 1 diabetes [37% (IQR 14–59); *n* = 4], and respiratory tract diseases [39% (IQR 20–60); *n* = 28].

**Table 1:** Median enrollment rates of women per different medical conditions examined in the included randomized controlled trials

Medical conditions	Number of trials (%)	Median enrollment rates of women, % (percentiles 25–75)
Viral diseases not including HIV/AIDS	9 (3)	42 (31–60)
HIV/AIDS	13 (4)	33 (9–57)
Bacterial and fungal infections	24 (8)	37 (25–49)
Cardiovascular diseases (overall)	85 (29)	39 (25–52)
Ischemic heart disease	17 (6)	22 (17–33)
Congestive heart failure	14 (5)	34 (25–53)
Stroke	15 (5)	41 (34–49)
Hypertension	11 (4)	38 (19–52)
Digestive system diseases	26 (9)	47 (33–54)
Liver disease	12 (4)	35 (26–46)
Neoplasms	53 (18)	39 (28–47)
Neoplasms not including lung cancer	44 (15)	41 (28–48)
Endocrine system diseases (overall)	39 (13)	46 (33–58)
Diabetes type 1	4 (1)	37 (14–59)
Diabetes type 2	36 (12)	46 (34–57)
Respiratory tract diseases	28 (10)	39 (20–60)
Immune system diseases	12 (4)	68 (46–81)

Trial characteristics and their association with median enrollment rates of women are presented in Table 2. The median enrollment rate of women decreased with older age of the trial’s participants [mean age of trials’ participants ≤ 45 years: 47% (IQR 30–64); *n* = 69, 46 to 55 years: 46% (IQR 33–58); *n* = 71, 56 to 62 years: 38% (IQR 27–50); *n* = 76, ≥ 63 years: 33% (IQR 20–46); *n* = 67, *p* < 0.001]. Studies testing hard primary outcomes had a lower enrollment rate than trials examining soft outcomes [35% (IQR 26–47); *n* = 34, compared with 43% (26–59); *n* = 130, *p* ≤ 0.001]. Trials of invasive interventions included significantly fewer women than those of non-invasive interventions [31% (IQR 22–46); *n* = 55, compared with 42% (29–58); *n* = 234, *p* < 0.001]. A third of the trials investigating invasive interventions (20/55) were in the field of cardiovascular diseases. There was no significant difference in the median enrollment rate of women between developed and developing countries [39% (IQR 25–54); *n* = 170, compared with 41% (IQR 28–54); *n* = 86, *p* = 0.448].

**Table 2:** Associations between enrollment rate and trial characteristics of the 289 included randomized controlled trials—univariate analysis

Trial characteristics	Number of trials, *n* (%)	Median enrollment rates of women, % (percentiles 25–75)	Univariate analysis *p*-value
Funding source			0.096
Industry	80 (28)	39 (27–51)	
Others	209 (72)	42 (27–58)	
Country classification			0.448
Developed economies	170 (59)	39 (25–54)	
Developed and developing economies	33 (11)	42 (32–54)	
Developing economies	86 (30)	41 (28–54)	
Age			< 0.001
Quartile 1 (≤ 45)	71 (24)	47 (30–64)	
Quartile 2 (46–55)	72 (25)	46 (33–58)	
Quartile 3 (56–62)	76 (26)	38 (27–50)	
Quartile 4 (≥ 63)	70 (24)	33 (20–46)	
Type of intervention			< 0.001
Invasive	55 (19)	31 (22–46)	
Non-invasive	234 (81)	42 (29–58)	
Type of comparison			0.357
Drug vs. drug	95 (33)	38 (24–50)	
Drug vs. placebo	63 (22)	42 (29–58)	
Others	131 (45)	44 (28–54)	
Setting			0.898
Inpatients	55 (19)	37 (27–46)	
Outpatients	234 (81)	42 (27–58)	
Type of primary outcome			< 0.001
Hard	34 (12)	35 (26–47)	
Surrogate	125 (43)	38 (23–53)	
Soft	130 (45)	44 (30–59)	
Adequate sequence generation	211 (73)	42 (27–58)	0.113
Allocation concealment	189 (65)	42 (28–59)	0.188
Blinding			0.135
Single or no blinding	181 (63)	40 (27–53)	
Double or triple	108 (37)	42 (27–56)	

Methodological quality characteristics (allocation concealment, blinding of participants, blinding of trial personnel) showed no significant association with the median enrollment rates of women.

Out of the 300 included RCTs, eleven did not report on the number of included women. The characteristics of these studies are presented in Additional file 3: Table S1. There was no significant difference between these studies and the studies included in the analysis.

## Discussion

The overall median enrollment rate of women was 41%. Older age of study participants, invasive interventions (mostly in trials assessing cardiovascular diseases), and studies with hard primary outcomes were related to lower women’s enrollment rates. Methodological quality characteristics showed no significant association with median enrollment rates of women. The association between older age and lower enrollment rates of women has been previously described by Vitale et al. [21, 22]. This population of older women represents a large proportion of real-world drug and treatment recipients. Unfortunately, their underrepresentation creates an absence of crucial data for the estimation of the interventions’ safety, adverse events, and real-world effectiveness [21, 22].

When looking specifically at women’s enrollment rates by medical conditions, the underrepresentation of women in cardiovascular diseases, HIV, stroke, and cancer is in line with the findings published in previous systematic reviews in these fields [10–16]. However, we have observed low enrollment rates of women in trials for type 1 diabetes mellitus and bacterial and fungal infections. We have looked at several large cohort studies in these fields which reflect the target populations. In the Pittsburgh Epidemiology of Diabetes Complications Study, which included a large cohort of young US adults with type 1 diabetes mellitus, the proportion of women was 49% (compared to a median of 37% in the trials included here) [23]. Another large cohort included 4306 clinically diagnosed adult patients with type 1 diabetes mellitus attending the outpatient clinic at Steno Diabetes Center in Gentofte, Denmark, from 2001 to 2013. The proportion of women was 46% in this cohort and 42% in the validation cohort for this study (*n* = 2118) [24].

We also examined large observational cohorts in the field of bacterial and fungal infections covering most of the topics included in our systematic review: hospital-acquired surgical site infection (SSI) [25], *Clostridioides difficile* infection (CDI) [26], catheter-associated urinary tract infection (CA UTI) [27], and neutropenic fever (NF) [28]. The percentage of women in these cohorts was 42–54%, higher than the rate we observed in randomized trials (37%).

Explanations suggested for low recruitment rates of young women in RCTs were fluctuations in the female hormones, which could affect the outcome of the intervention, adding more variability to the data, and concerns regarding exposing women with reproductive potential, pregnant or lactating, to experimental drugs [3]. Moreover, including a woman with child-bearing potential in a study usually requires sampling of serum or urine for β-hCG and conducting a contraception check, which complicates the recruitment process, skewing recruitment towards men. For post-menopausal women, one explanation for low recruitment rates is that women suffer more from dementia [29], which might complicate the consent procedures.

There are potentially other, unmeasured characteristics that could account for this gap, especially ones that are related to cultural barriers: low literacy levels compared to men (interfering in the informed consent process), modesty, the fear of stigma that causes women to seek less help, or discrimination in health care service utilization [29]. Another explanation could be that in different regions, especially in developing countries, women are not the predominant decision-makers in matters concerning their health [30].

A limitation of our study is the exclusion of non-English papers (*n* = 10). In these trials, women’s enrollment rates might be even lower. Another limitation is that we did not collect data on women’s retention rates from RCTs. In some studies, women were more likely to prematurely discontinue the study drug and withdraw consent from the trial compared to men [31, 32]. It could be of value to study women’s retention rates from RCTs and to demonstrate the rates of missing information for this outcome.

Factors that might hinder the participation of women should be sought and addressed in the design of the study. The proportion of included women can be estimated at the protocol writing stage and followed during the trial. Strategies to improve the participation of women in RCTs should be implemented: improvement of the explanation about the benefits of the trial, to dispel potential misconceptions, and obtaining feedback from both men and women, who declined to participate, to better understand potential barriers to enrollment of women. An important action towards improving gender equity in medical research is to ensure that the study leadership, including the study executive committee and site investigators, includes both men and women. Nielsen et al. showed a robust positive correlation between women’s authorship and the likelihood of a study including gender and sex analysis [33].

In conclusion, we found that women are being inadequately represented, in the selected medical fields analyzed in our study, in recent RCTs. Older age is a potential barrier to enrollment of women in clinical trials. Low inclusion rates of elderly women in clinical trials might create a lack of crucial knowledge of the adverse effects and the benefit/risk profile of any given treatment. Reporting sex-stratified outcomes for both efficacy and adverse events is of high importance. RCT investigators should increase their efforts to recruit women who are eligible for enrollment so that their proportion in the study sample will be as close to the real-life population as possible.

## Supplementary Information

**Additional file 1:** **Appendix 1.** Search phrase.

**Additional file 2.** PRISMA Checklist.

**Additional file 3: Table S1.** Characteristics of studies that did not report the number of included women and their comparison to studies that report the number of included women.

## Data Availability

No additional data is available.

## Abbreviations

CA UTI: Catheter-associated urinary tract infection
SSI: Surgical site infection
CDI: *Clostridium difficile* infection
CR GNB: Carbapenem-resistant Gram-negative bacteria
CONSORT: Consolidated Standards of Reporting Trials
EU: European Union
FDA: Food and Drug Administration
GLM: Generalized linear models
GAO: Government Accountability Office
HIV: Human immunodeficiency virus
IND: Investigational new drug
IQR: Interquartile range
MESH: Medical Subject Headings
PRISMA: Preferred Reporting Items for Systematic Reviews
RCT: Randomized controlled trial
SPSS: Statistical Package for the Social Sciences
