# Supplementary material for: Underrepresentation of women in randomized controlled trials: a systematic review and meta-analysis
Source: Trials. 2022 Dec 21;23:1038. doi: 10.1186/s13063-022-07004-2 (PMC9768985; doi:10.1186/s13063-022-07004-2)
Supplement: Supplementary file 1 — Additional file 1: Appendix 1. Search phrase. [file 13063_2022_7004_MOESM1_ESM.docx]

Supplemental Appendix 1: Search phrase

((((randomized controlled trial [pt] OR controlled clinical trial [pt] OR randomized [tiab] OR placebo [tiab] OR clinical trials as topic [mesh: noexp] OR randomly [tiab] OR trial [ti]) NOT (animals [mh] NOT humans [mh])))) **AND** ("Bacterial Infections and Mycoses"[Mesh] OR "Virus Diseases"[Mesh] OR "Neoplasms"[Mesh] OR "Digestive System Diseases"[Mesh] OR "Respiratory Tract Diseases"[Mesh] OR "Cardiovascular Diseases"[Mesh] OR "Endocrine System Diseases"[Mesh] OR "Immune System Diseases"[Mesh]).
