# Supplementary material for: Underrepresentation of women in randomized controlled trials: a systematic review and meta-analysis
Source: Trials. 2022 Dec 21;23:1038. doi: 10.1186/s13063-022-07004-2 (PMC9768985; doi:10.1186/s13063-022-07004-2)
Supplement: Supplementary file 3 — Additional file 3: Table S1. Characteristics of studies that did not report the number of included women and their comparison to studies that report the number of included women. [file 13063_2022_7004_MOESM3_ESM.docx]

Supplementary table 1: characteristics of studies that did not report the number of included women and their comparison to studies that report the number of included women.

| Trial characteristics | Studies that did not report the number of included women  N= 11 (%) | Studies that report the number of included women  N= 289 (%) | Univariate analysis P-value |
| --- | --- | --- | --- |
| Funding |  |  | 0.733 |
| Industry | 2 (18) | 80 (28) |  |
| Other | 9 (82) | 209 (72) |  |
| Country classification |  |  | 0.333 |
| Developed economies | 4 (36) | 170 (59) |  |
| Developed and Developing economies | 2 (18) | 33 (11) |  |
| Developing economies | 5 (46) | 86 (30) |  |
| MESH |  |  | 0.618 |
| Bacterial and fungal infections | 3 (27) | 24 (8) |  |
| Virus diseases | 1 (9) | 22 (8) |  |
| Neoplasms | 2 (18) | 53 (18) |  |
| Digestive system diseases | 1 (9) | 26 (9) |  |
| Respiratory tract diseases | 1 (9) | 28 (10) |  |
| Cardiovascular diseases | 2 (18) | 85 (29) |  |
| Endocrine system diseases | 1 (9) | 39 (13) |  |
| Immune system diseases | 0 (0) | 12 (4) |  |
| Intervention procedure |  |  | 0.450 |
| Invasive | 3 (27) | 55 (19) |  |
| Noninvasive | 8 (73) | 234 (81) |  |
| Setting - patients |  |  | 0.450 |
| Inpatients | 3 (27) | 55 (19) |  |
| Outpatients | 8 (73) | 234 (81) |  |
| Allocation concealment |  |  | 0.525 |
| Low | 6 (55) | 189 (65) |  |
| High | 5 (45) | 100 (35) |  |
| Blinding |  |  | 0.459 |
| Single or no blinding | 8 (73) | 181 (63) |  |
| Double or triple | 3 (27) | 108 (37) |  |
| Intervention type |  |  | 0.711 |
| Drug vs. drug | 8 (73) | 226 (77) |  |
| Drug vs. placebo | 3 (27) | 63 (23) |  |
| Outcome type |  |  | 0.759 |
| Hard | 1 (9) | 34 (12) |  |
| Soft | 4 (36) | 125 (43) |  |
| Surrogate | 6 (55) | 130 (45) |  |
| Age |  |  | 0.722 |
| Age (med< 56) | 4 (57)  N=7 | 140 (48)  N=283 |  |
| Age (med>=56) | 3 (43)  N=7 | 143 (52)  N=283 |  |
